# Microstructure and Friction Properties of AlCrTiVNb_x_ High-Entropy Alloys via Annealing Manufactured by Vacuum Arc Melting

**DOI:** 10.3390/ma17040812

**Published:** 2024-02-08

**Authors:** Baowei Li, Zihao Zhang, Xiaoling Luo, Kangmin Chen, Jiaqi Zhang, Pan Gong, Zhen Peng

**Affiliations:** 1 School of Materials Science and Engineering, Jiangsu University, Zhenjiang 212013, China; 2 State Key Laboratory of Materials Processing and Die & Mould Technology, School of Materials Science and Engineering, Huazhong University of Science and Technology, No. 1037 Luoyu Road, Wuhan 430074, China; 3 Research Institute of Huazhong University of Science and Technology in Shenzhen, Shenzhen 518057, China

**Keywords:** high-entropy alloy, annealing treatment, frictional wear

## Abstract

To enhance the friction and wear properties of alloys, AlCrTiVNb_x_ high-entropy alloys (HEAs) with various Nb contents were prepared using the arc melting technique and then annealed at 1000 °C for 2 h. The microstructure and hardness changes in the AlCrTiVNb_x_ (x = 0.3, 0.4, and 0.5) HEAs after casting and annealing were studied via scanning electron microscopy, X-ray diffractometry, optical microscopy and the Vickers hardness test. The MFT-EC400 ball disc reciprocating friction and wear tester was used to investigate the wear resistance of the HEAs before and after annealing. The results show that the annealed AlCrTiVNb_x_ HEAs changed from a single-phase structure to a multi-phase structure, and the content of the face-center cubic (FCC) phase and hexagonal close-packed (HCP) phase further increases with the increase in Nb content. The hardness value of the annealed HEAs is greatly enhanced compared with the casting state, and the hardness of the Nb_0.5_ HEA is increased from 543 HV to 725 HV after annealing. The wear resistance of the alloys after the annealing treatment is also greatly improved, among which Nb_0.5_ has the best wear resistance. The average friction coefficient of Nb_0.5_ is 0.154 and the wear rate is 2.117 × 10^−5^ mm^3^/(N·m). We believe that the precipitation strengthening after the annealing treatment and the lubrication effect of the FCC phase are the reasons for the significant improvement in wear resistance. The morphology of the samples indicates that the wear mechanism of the alloy includes adhesive wear, abrasive wear and a certain degree of oxidation wear.

## 1. Introduction

At the beginning of the 21st century, Yeh [1] and Cantor [2] first proposed the concept of a multi-principal element alloy, that is, a solid solution formed by mixing five or more elements according to a certain molar ratio (the atomic percentage of each element is between 5% and 35%), and defined it as a high-entropy alloy (HEA). HEAs have been shown to have a variety of outstanding properties, such as high strength values, high hardness values, and excellent corrosion resistance and wear resistance [3,4,5,6,7,8,9,10,11,12], leading to extensive research and development as well as high application potential. More specifically, HEAs are expected to be applied as high temperature-resistant alloys, wear-resistant alloys and corrosion-resistant alloys, etc. Unlike conventional alloys, which are generally composed of one or two primary elements, HEAs possess a plethora of unique characteristics that place them at the central region of the phase diagram. Due to their extensive alloy composition space and wide-ranging microstructure possibilities, HEAs exhibit remarkable potential for use in the field of friction materials [13,14,15,16,17,18,19,20,21]. 

There have been a series of studies on improving the wear resistance of HEAs. The highest hardness values of Al_x_CoCrFeNi (x = 0.3–1.0), Al_x_CoCrFeMnNi (x = 0–2.0), FeCoCrNiMnAl_x_ (x = 0–1.0) and CoCrFeNiNb_x_ (x = 0.5–0.8) are more than 600 HV. Among these, the friction coefficients of FeCoCrNiMnAl_x_ (x = 0–1.0) are below 0.3 [22,23,24,25]. Al_2_CrFeCoCuTiNi_x_ (x = 0–2.0), FeCoNiCrSiAl_x_ (x = 0–1.5) and FeCoCrNiAlTi_x_ (x = 0–1.0) have the highest hardness values, which are more than 900 HV [26,27,28]. The isoatomic AlCrTiV HEA has the characteristics of a single-BCC (B2) solid solution, with a relatively low density of 5.06 g/cm^3^ and a hardness value of 498 HV. The theoretical density of AlCrTiV-Si_5_ alloys is between 4.7 and 4.9 g/cm^3^, and the microhardness can reach an extremely high Vickers hardness of 679 HV_0.2_ [29]. As a result, HEAs are currently reported to be one of the alloys with the highest specific hardness values [30].

Feng et al. [31] prepared AlCrFeNiV HEAs by spark plasma sintering, consisting of two different types of body-centered cubic BCC structures with Vickers hardness values of more than 1000 HV. Chauhan et al. [32] prepared Al_35_Cr_14_Mg_6_Ti_35_V_10_ HEAs by spark plasma sintering and recorded a hardness of 460 HV, which is higher than that of other Al-, Mg- and Ti-based conventional alloys. The hardness of AlCrTiVCu_0.2_ HEAs prepared by Peng et al. [33] reached 814 HV, higher than that of most conventional metal alloys. In order to improve the friction and wear properties of Ti-6Al-4V, Hao et al. [34] coated the Ti-6Al-4V matrix with AlTiVCrNb HEAs via laser cladding. The microhardness of the coating is 1.57 times that of the matrix, and the wear resistance is 1.58 times higher than that of the matrix. Nussbaum et al. [35] prepared HEA nitride (AlCrTiV) N coatings by the cathode arc evaporation method. The film deposited at 100 V and 300 °C has good mechanical properties, with a hardness value of 40 GPa, friction coefficient of 0.44 and wear coefficient of 5.8 × 10^−7^ mm^3^·N^−1^m^−1^. Therefore, it is considered to be an effective method to improve the microhardness and wear resistance by selectively adjusting various elements in HEAs, thereby changing the existing phase in the HEAs.

At present, there are generally three ways to improve the wear resistance of HEAs [5,36,37,38,39,40]. Firstly, the addition of modified metal elements can improve the properties of HEAs, such as chemically active metal elements like Al and Ti, and metal elements with high melting points, such as Nb, W, etc. This method primarily employs chemical reaction enthalpy as a means to reinforce the lattice distortion of the alloy. This technique induces the formation or precipitation of the reinforcing phase and the lubricating phase and exerts an influence on the oxide layer and alloy microstructure morphology, thereby achieving the aim of enhancing the properties of HEAs, for instance, Nb alloys of CoCrFeNi HEAs [41,42]. The incorporation of niobium atoms not only exacerbates the lattice distortion of the alloy, resulting in the phenomenon of solid-solution strengthening, but also lowers the mixing enthalpy between niobium and the matrix atoms, which engenders the facile formation of intermetallic compounds and consequent secondary-phase strengthening, thereby ameliorating the holistic properties of the HEAs. Moreover, the surface structure of the alloy is improved by the heat treatment process [43]. The main manifestations of the heat treatment process are as follows: (1) an effect on the degree of lattice distortion and the content proportion of the phase in the alloy; (2) the precipitation or dissolution of the enhanced phase; and (3) grain coarsening. The as-cast AlCoCrFeNiTi_0.5_ HEA is predominantly composed of a higher proportion of the B2 phase and a minor A2 phase [44]. Upon undergoing annealing at 800 °C for 5 h, the alloy experiences significant lattice distortion, leading to an increase in the intergranular phase. Additionally, the alloy exhibits improved resistance to dislocation at grain boundaries, resulting in a 26% increase in its hardness and enhanced wear resistance. The third way is to modify its surface structure through surface engineering technology [45], such as carburizing, nitriding and boronizing. For example, with Ni_45_(CoCrFe)_40_(AlTi)_15_ surface nitriding [46], AlN, CrN and TiN phases are formed on the surface, effectively increasing the surface hardness of the alloy from 8.8 GPa to 14.9 GPa. Furthermore, the utilization of shot peening, laser remelting and various other technologies also have the potential to enhance the surface characteristics of HEAs [47,48].

Al, Ti and Cr are widely used in alloys because of their effects on microstructure, strengthening and oxidation resistance. V is commonly used to improve friction and wear properties because its atomic size deforms the crystal structure of BCC and FCC alloys. As a lightweight alloy, the mechanical properties and corrosion resistance of the HEA of AlCrVTi have been studied, but the effect of the content of Nb on the wear resistance of the HEA has not been studied [49,50,51,52]. In this paper, AlCrTiVNb_x_ (x = 0.3, 0.4, 0.5) HEAs were prepared by vacuum arc melting and annealed at 1000 °C for 2 h to investigate the changes in microstructure and mechanical properties before and after annealing. Moreover, dry friction wear tests were conducted to investigate the impact of heat treatment on the friction and wear performance of the HEAs.

## 2. Experimental Methods

In the present study, ingots of HEAs with the predetermined compositions of AlCrTiVNb_x_ (x = 0.3, 0.4,and 0.5) were prepared in a non-self-consumable vacuum arc melting furnace (AM-800-3, Shenzhen KeJing, Shenzhen, China) using pure metals (with purity greater than 99.9 wt%). The metal raw materials were placed in copper crucibles according to the melting point and melted 5 times to obtain a homogeneous structure. The alloy ingot was annealed at a temperature of 1000 °C for 2 h using a muffle furnace at heating speed of 10 °C/min, and then the furnace was cooled to room temperature after heat preservation.

The as-cast HEAs were cut into the dimensions of 10 mm × 10 mm × 3 mm, and then the surface was polished using sandpapers to measure Vickers hardness using HV-1000IS (SIOMM, Shanghai, China) micro Vickers hardness tester.

The phase structures were characterized by X-ray diffraction (XRD, Rigaku SMARTLAB9, Bright Industrial (Shanghai) Co., LTD., Shanghai, China) equipment with the accelerating voltage of 40 kV. The microstructure and elemental composition of AlCrTiVNbx HEAs were investigated via scanning electron microscopy (SEM, FEI Nova Nano450, Thermo Fisher Scientific, Shanghai, China) with energy-dispersive X-ray spectrometer (EDS, Thermo Fisher Scientific, Shanghai, China) detector for elemental analysis. The wear tests for the AlCrTiVNbx HEAs were conducted using a Lanzhou Huahui MFT-EC400 tribometer (HuahuiInstrument Technology Co., Ltd., Lanzhou, China) at room temperature in the reciprocating mode using GCr15 stainless-steel ball of φ5 mm, and the experimental parameters were an oscillating amplitude of 5 mm, normal load of 5 N, frequency of 2 Hz and sliding time of 30 min. To ensure the accuracy of the results, wear tests with the same experimental parameters were carried out at least three times. After wear tests, the three-dimensional profiles of wear tracks were obtained via Leica light microscopy, and the SEM micrographs of the wear tracks were analyzed via FEI scanning electron microscopy. Then, the wear rate and average friction coefficient were calculated.

## 3. Results and Discussion

### 3.1. Microstructural Investigations

**Figure 1:**
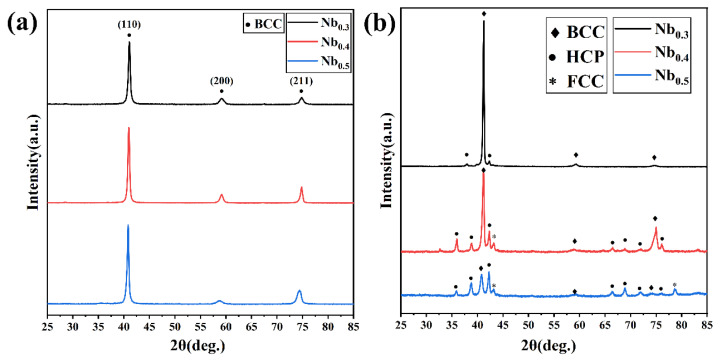
XRD patterns of AlCrTiVNb_x_ (x = 0.3, 0.4, 0.5) HEAs before and after annealing; (**a**) As-cast and (**b**) Annealed.

Figure 1 shows the XRD patterns of the as-cast and annealed AlCrTiVNb_x_ (x = 0.3, 0.4, and 0.5) HEAs. It can be seen from Figure 1a that the AlCrTiVNb_x_ HEA exhibits a single BCC phase in the initial casting state. After annealing at 1000 °C for 2 h, the AlCrTiVNb_0.3_ HEA depicts a duplex structure of the BCC phase and HCP phase. As the Nb content further increased to 0.4 and 0.5, the XRD patterns of AlCrTiVNb_0.4_ and AlCrTiVNb_0.5_ showed more diffraction peaks, and the peaks of the FCC phases appeared. According to the standard JCPDS PDF cards (PDF Card 01-074-5286, 04-003-9781, 00-007-0281), the BCC phase is the Al_0.5_Ti_0.5_V phase rich in Ti and V elements, the HCP phase is the NbCrAl phase rich in Nb and Cr elements and the FCC phase is the Al_11_V phase rich in Al and V. After annealing, the diffraction peak intensity of the BCC phase decreases and the number of diffraction peaks of the HCP phase increases, indicating that the increase in Nb content is conducive to the formation of the HCP phase after annealing.

**Figure 2:**
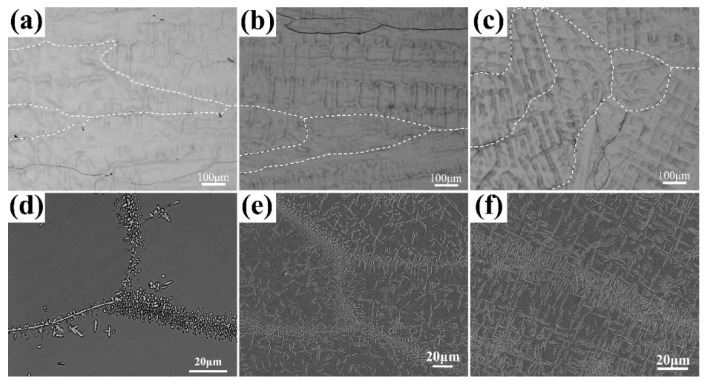
Microscopic morphology of as-cast AlCrTiVNb_x_ (x = 0.3, 0.4 and 0.5) before and after annealing; (**a**) Nb_0.3_, (**b**) Nb_0.4_, (**c**) Nb_0.5_, annealed, (**d**) Nb_0.3_, (**e**) Nb_0.4_ and (**f**) Nb_0.5_.

Figure 2a–c shows the SEM morphologies of the as-cast AlCrTiVNbx (x = 0.3, 0.4, 0.5) HEAs, and the annealed samples are shown in Figure 2d–f. It can be seen from the figures that the as-cast AlCrTiVNb_x_ HEAs present a similar morphology with an elongated columnar crystal structure and coarse grains, and they have a dendritic appearance after enlargement. After annealing at 1000 °C for 2 h, the annealed AlCrTiVNb_x_ HEAs exhibited an amplitude modulation decomposition structure and generated a fine precipitated phase. When the Nb content is low, the precipitated phase is mainly concentrated near the grain boundary, showing a point-like structure. When the Nb content further increases, the precipitated phase gradually diffuses from the grain boundary to the entire grain. But the precipitated phase at the grain boundary is smaller and denser, while the precipitated phase inside the crystal presents a needle-like structure.

**Figure 3:**
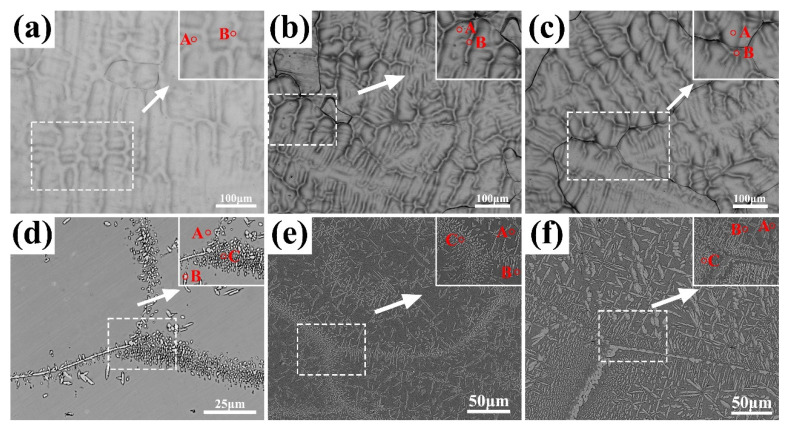
Composition selection diagram of as-cast AlCrTiVNb_x_ (x = 0.3, 0.4 and 0.5) HEAs before and after annealing; (**a**) Nb_0.3_, (**b**) Nb_0.4_, (**c**) Nb_0.5_ annealed, (**d**) Nb_0.3_, (**e**) Nb_0.4_ and (**f**) Nb_0.5_.

The phase composition of AlCrTiVNbx HEAs was further analyzed using EDS, and the results are shown in Figure 3, Table 1 and Table 2. Figure 3a–c show the composition selection point diagram of the as-cast high-entropy alloys. Figure 3d–f show the annealed samples. Table 1 and Table 2 shows the nominal and actual composition of the AlCrTiVNb_x_ HEAs. It can be seen from Table 1 that the composition of the cast AlCrTiVNb_x_ HEAs is close to their nominal composition, and the element distribution is uniform with no obvious element segregation.

After annealing at 1000 °C for 2 h, a composition analysis of the AlCrTiVNb_x_ HEAs was carried out, and the results are shown in Table 2. It can be seen from Table 2 that the content of the Al, Ti and V elements in the matrix is higher than that of the precipitated phase, while the content of Cr and Nb in the precipitated phase is higher. Compared with the XRD pattern results, the matrix is the Al_0.5_Ti_0.5_V phase and the precipitated phase is the NbCrAl phase.

### 3.2. The Hardness of the Alloy

**Figure 4:**
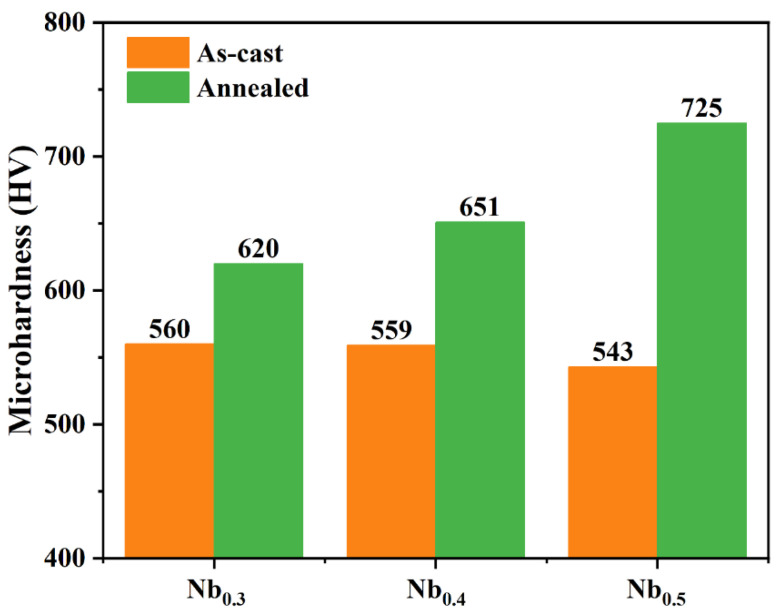
Microscopic Vickers hardness before and after annealing of AlCrTiVNb_x_ HEAs.

Figure 4 shows the microscopic Vickers hardness of the AlCrTiVNb_x_ HEAs before and after annealing. It is evident that the hardness of the as-cast AlCrTiVNb_x_ HEAs experiences a marginal decline with the increase in Nb content. This phenomenon can be attributed to the decrease in the atomic radius difference of the alloy, which in turn leads to a reduction in the lattice malformation of the alloy. Consequently, the hardness of the alloy exhibits a slight decrease. After annealing, the hardness of the AlCrTiVNb_x_ HEAs increased, among which the hardness value of Nb_0.5_ is the highest, reaching 725 HV. This may be due to the fact that after annealing, with the increase in the Nb content, the content of the NbCrAl precipitated phase also increases, and more precipitated phases play a role in precipitation strengthening. Thus, the hardness of the AlCrTiVNb_x_ HEAs after annealing is significantly improved.

### 3.3. Friction and Wear Property

**Figure 5:**
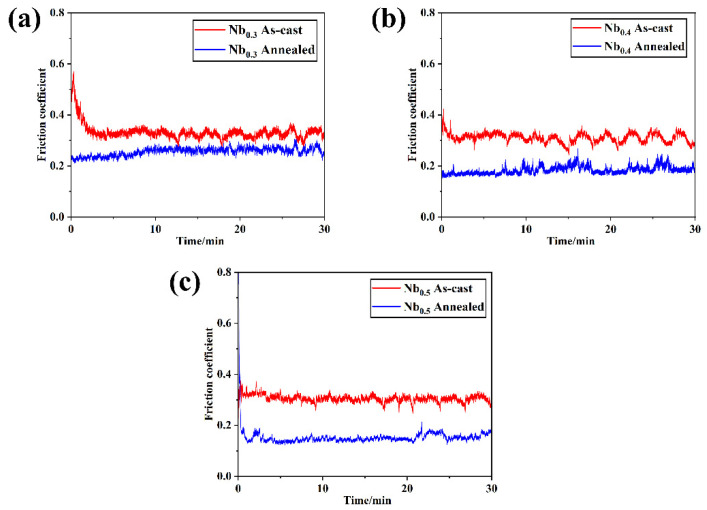
Friction coefficient before and after annealing of AlCrTiVNb_x_ HEAs; (**a**) Nb_0.3_, (**b**) Nb_0.4_ and (**c**) Nb_0.5_.

Figure 5 shows the curve of the friction coefficient (f) with the wear time (t) obtained by the normal application of a 5 N load to the grinding ball of AlCrTiVNb_x_ (x = 0.3, 0.4 and 0.5) HEAs before and after annealing with GCr15 under atmospheric conditions. After the friction starts, the friction coefficient fluctuates greatly in the first 5 min, and there is a run-in stage between the sample surface and the grinding head. The friction coefficient fluctuation tends to flatten out after 5 min of the wear experiment. It can be seen from the figure that the friction coefficient of the cast AlCrTiVNb_x_ HEAs is between 0.3 and 0.4, and the friction coefficient of the AlCrTiVNb_x_ HEAs after annealing has significantly decreased by around 0.1–0.3. We use the stage at which the fluctuation between 5 and 30 min is relatively flat, and the friction coefficient is averaged to obtain the average friction coefficient, as shown in Table 3. From Table 3, we can see that the average friction coefficient of the as-cast AlCrTiVNb_x_ HEAs increases slightly with the increase in Nb content: 0.305, 0.311 and 0.333, respectively. The average friction coefficient of the AlCrTiVNb_x_ HEAs after annealing is the opposite: 0.203, 0.187 and 0.154, respectively. Generally speaking, the wear resistance of the alloy often has a certain correlation with the hardness, that is, the higher the hardness of the alloy, the better its wear resistance, which is manifested in the lower friction coefficient. The change in the average coefficient of friction of the HEAs in the cast and annealed states is consistent with this law.

The reason for the improvement of the wear resistance of the annealed HEAs may be due to the increase in Nb element content and the increases in the NbCrAl precipitated phases. Moreover, they have a more even distribution in the matrix, which leads to precipitation strengthening. At the same time, according to the XRD pattern, it can be seen that the Al_11_V phase of the FCC structure appears in the HEAs of Nb_0.4_ and Nb_0.5_, and the FCC structure is prone to plastic deformation and plays a certain role in the lubrication process during the wear process. Compared with as-cast HEAs, annealed HEAs have higher hardness values and better wear resistance.

### 3.4. Morphlology and Wear Mechanism of Wear Scars

**Figure 6:**
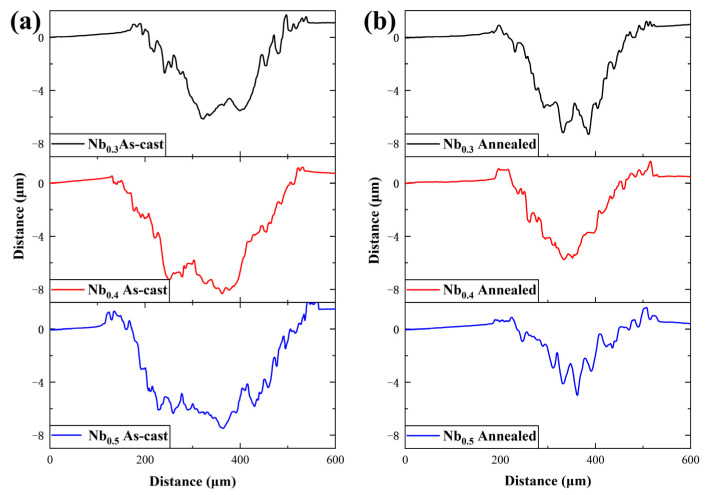
Wear scars section of AlCrTiVNbx HEAs after friction and wear tests: (**a**) as-cast and (**b**) annealed.

An Olympus OLS4100 laser confocal microscope (Olympus, Taiwan, China) was used to determine the width and depth of the wear scars after the friction and wear tests of the sample, and the results are shown in Figure 6. The wear scars’ volume and wear rate are calculated by the following formula:

$$W_{v}=\frac{Lh}{6b}\left(3h^{2}+4b^{2}\right)$$

$$K=\frac{W_{v}}{PS}$$

where *W_v_* is the volume of the wear scars in mm^3^; *b* is the width of the wear scars in μm; *h* is the depth of the abrasion scars in μm; *L* (5 mm) is the length of the wear scars; K is the wear rate in mm^3^/(Nm); *P* (5 N) is the applied load; and *S* is the total friction stroke in m.

From the test results in Table 4, it can be seen that the wear rate of the cast AlCrTiVNb_x_ HEAs is higher than that of the annealed state, among which the annealed AlCrTiVNb_0.5_ HEAs have the lowest wear rate of 2.117 × 10^−5^ mm^3^/(N·m), which shows the best wear resistance compared with that of the cast AlCrTiVNb_0.5_ HEAs. Their wear resistance is improved by 62.9%.

The friction and wear processes of materials are complex processes of mechanical and chemical coupling. Under the action of a variety of different forces between the alloy and the friction pair, the surface of the alloy is damaged, peeled, etc. At present, the classification of wear mainly includes the following: abrasive wear, adhesive wear, surface fatigue wear and corrosion wear.

**Figure 7:**
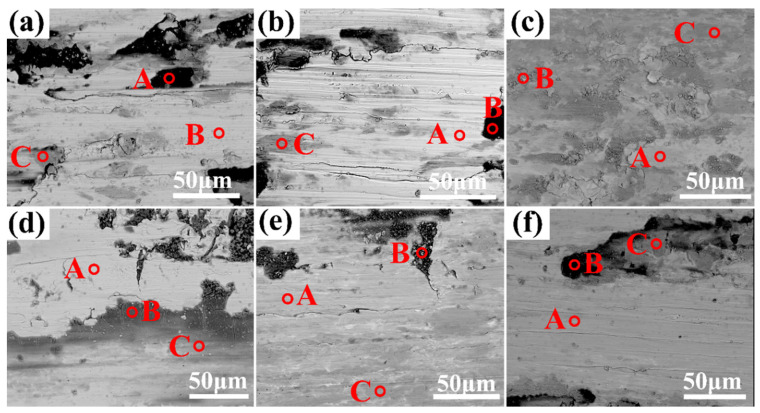
Surface morphology of as-cast AlCrTiVNbx HEAs after friction and wear tests. (**a**) Nb_0.3_, (**b**) Nb_0.4_, (**c**) Nb_0.5_ Annealed, (**d**) Nb_0.3_, (**e**) Nb_0.4_ and (**f**) Nb_0.5_.

Figure 7 shows the surface morphology of the AlCrTiVNb_x_ HEAs under dry friction after the friction and wear tests. Figure 7a–c show the surface morphology of as-cast HEAs after the wear test, and Figure 7d–f show it after they are annealed. It can be seen from Figure 7 that there is a furrow on the surface of the alloy that is obviously parallel to the sliding direction, which is caused by the contact of tiny particles with the surface during friction, which indicates that the alloy has abrasive wear during the wear process. In Figure 7a–f, there are more irregular sheet protrusions because in the friction process, Al, Ti, Cr and other elements generate oxide film. With the continuation of friction, the oxide film and the matrix with an loose combination produce cracks and fall off. When the oxide layers fall off into abrasive chips, these abrasive chips and the abrasive chips generated by the adhesive wear together produce the groove in the figure, exposing the matrix. Meanwhile, from Table 5, it can be seen that the black raised flakes in the B region of the alloy all contain more than 60% O elements, mainly the oxides of Al and Cr, so it can be proven that there is oxidative wear. A in the figure shows the matrix part of the alloy, and the EDS results show that the content of the O element in the matrix is around 40%. This indicates that during the wear process, the matrix produces a layer of oxide film, and at the same time, the surface oxide film of the alloy in the annealed state is more continuous and dense compared to that in the as-cast state. From Figure 7c, it can be seen that the wear surface of the cast Nb_0.5_ is more severe than that of the other alloys, with obvious surface detachment and a loose structure. From the EDS, it can be seen that the O content on the surface is higher than that in the other samples, which indicates that this sample has experienced more severe oxidative wear during the frictional wear process. Combined with the above analysis, it can be seen that the alloy has a wear mechanism of abrasive wear, adhesive wear and oxidation wear in the process of friction and wear.

## 4. Conclusions

(1) AlCrTiVNb_x_ (x = 0.3, 0.4, 0.5) HEAs have a BCC single-phase structure when it comes to casting, and the alloys undergo amplitude modulation decomposition after annealing treatments at 1000 °C for 2 h. They form a similar network structure, of which Nb_0.3_ has a BCC and HCP duplex structure, and Nb_0.4_ and Nb_0.5_ have a BCC, HCP and FCC three-phase structure.
(2) The hardness of the HEAs after the annealing treatment is greatly increased compared to that in the casting state, and the increase is more obvious with the increase in Nb content, among which the alloy hardness of Nb_0.5_ is the most obvious, increasing from 543 HV in the casting state to 725 HV. The reason for the increase in hardness is the increase in Nb content, the higher content of the HCP phase and BCC phase precipitated in the alloy and the combined effects of amplitude modulation decomposition and precipitation strengthening.
(3) The friction and wear properties of HEAs after the annealing treatment are significantly improved, and the annealed Nb_0.5_ sample has excellent wear resistance. It has an average friction coefficient of 0.154, and the wear rate is 2.117 × 10^−5^ mm^3^/(N·m). This is because the FCC phase and HCP phase produced by amplitude modulation decomposition after the annealing treatment play a role in precipitation strengthening, and the FCC phase has good plasticity and plays a certain role in self-lubrication.
(4) From the analyses of the post-wear SEM images and EDS results, it can be seen that the wear mechanism of the alloys in the annealed and as-cast states during the frictional wear process is the coexistence of abrasive wear, adhesive wear and oxidative wear, out of which the oxidative wear of the as-cast Nb_0.5_ is the most severe.

## Figures and Tables

**Table 1:** Distribution of chemical composition by region of as-cast AlCrTiVNb_x_ HEAs (at.%).

		Al	Cr	Ti	V	Nb
Nb_0.3_	A	25.0	22.3	21.3	23.1	8.4
	B	24.0	23.6	22.4	21.7	8.3
Nb_0.4_	A	23.8	21.5	21.4	22.7	10.9
	B	23.8	21.4	20.9	22.9	11.0
Nb_0.5_	A	22.6	21.1	21.0	21.9	13.3
	B	22.8	20.9	20.8	22.2	13.3

**Table 2:** Distribution of chemical composition of annealed AlCrTiVNb_x_ HEAs (at.%).

		Al	Cr	Ti	V	Nb
Nb_0.3_	A	25.2	22.0	22.4	22.2	8.2
	B	24.5	27.7	19.1	17.0	11.7
	C	22.5	29.2	19.0	17.4	11.9
Nb_0.4_	A	25.3	21.9	23.1	21.6	8.1
	B	22.1	29.5	19.0	17.4	12.0
	C	21.9	30.5	18.6	15.8	13.2
Nb_0.5_	A	25.3	21.9	23.1	21.6	8.1
	B	21.9	30.5	18.6	15.8	13.2
	C	22.1	29.4	19.0	17.4	12.1

**Table 3:** Average coefficient of friction before and after annealing of AlCrTiVNb_x_ HEAs.

Sample		Average Friction Coefficient
Nb_0.3_	As-cast	0.305
	Annealed	0.203
Nb_0.4_	As-cast	0.311
	Annealed	0.187
Nb_0.5_	As-cast	0.333
	Annealed	0.154

**Table 4:** Friction and wear parameters of AlCrTiVNb_x_ (x = 0.3, 0.4 and 0.5) samples.

		b (µm)	h (µm)	W_v_ (mm^3^)	K (mm^3^/(Nm))
As-cast	Nb_0.3_	367.5	6.14	0.0180	5.010 × 10^−5^
	Nb_0.4_	401.9	8.30	0.0192	5.337 × 10^−5^
	Nb_0.5_	442.5	7.48	0.0205	5.710 × 10^−5^
Annealed	Nb_0.3_	323.7	7.16	0.0123	3.404 × 10^−5^
	Nb_0.4_	300.6	5.76	0.0095	2.630 × 10^−5^
	Nb_0.5_	304.4	4.97	0.0076	2.117 × 10^−5^

**Table 5:** EDS analysis of wear marks of AlCrTiVNb_x_ (x = 0.3, 0.4, 0.5) high-entropy alloys after frictional wear tests.

			Al	Cr	Ti	V	Nb	O
As-cast	Nb_0.3_	A	17.71	14.84	14.25	16.41	5.75	31.04
		B	7.83	5.99	6.51	6.01	2.36	71.67
		C	13.39	11.28	11.33	11.42	4.33	48.25
	Nb_0.4_	A	17.12	16.42	16.24	16.47	7.94	25.81
		B	10.53	8.79	8.87	9.18	4.54	58.08
		C	15.21	13.79	13.37	13.79	6.73	37.48
	Nb_0.5_	A	11.48	11.06	10.73	10.64	6.62	49.47
		B	5.99	9.03	5.64	5.69	3.31	70.34
		C	6.96	8.38	6.71	6.73	3.93	67.30
Annealed	Nb_0.3_	A	14.82	13.54	13.15	13.67	5.03	39.78
		B	4.84	7.48	4.47	4.54	1.99	76.69
		C	11.08	10.85	10.61	10.85	3.92	52.69
	Nb_0.4_	A	14.64	13.60	13.30	14.00	6.67	37.71
		B	5.79	8.89	6.76	7.01	2.88	68.67
		C	8.73	8.95	8.66	8.79	3.96	60.91
	Nb_0.5_	A	15.92	12.18	13.39	13.11	7.72	37.68
		B	8.17	7.44	7.28	7.31	4.41	65.39
		C	14.29	13.97	13.54	13.83	8.26	36.11

## Data Availability

Data are contained within the article.

## References

[1] Yeh J.W., Chen S.K., Lin S.J., Gan J.Y., Chin T.S., Shun T.T., Tsau C.H., Chang S.Y. (2004). Nanostructured High-Entropy Alloys with Multiple Principal Elements: Novel Alloy Design Concepts and Outcomes. Adv. Eng. Mater..

[2] Cantor B., Chang I.T.H., Knight P., Vincent A.J.B. (2004). Microstructural development in equiatomic multicomponent alloys. Mater. Sci. Eng. A.

[3] Otto F., Dlouhý A., Somsen C., Bei H., Eggeler G., George E.P. (2013). The influences of temperature and microstructure on the tensile properties of a CoCrFeMnNi high-entropy alloy. Acta Mater..

[4] George E.P., Raabe D., Ritchie R.O. (2019). High-entropy alloys. Nat. Rev. Mater..

[5] Mousavi S.E., He A.Q., Palimi M., Chen D.L., Li D.Y. (2023). Influences of alloying elements on microstructure and tribological properties of a medium-weight high-entropy alloy. Wear.

[6] Miracle D.B., Senkov O.N. (2017). A critical review of high entropy alloys and related concepts. Acta Mater..

[7] Peng Z., Sun J., Luan H.W., Chen N., Yao K.F. (2023). Effect of Mo on the high temperature oxidation behavior of Al_19_Fe_20_-xCo_20_-xNi_41_Mo_2x_ high entropy alloys. Intermetallics.

[8] Tang Y., Wang R., Xiao B., Zhang Z., Li S., Qiao J., Bai S., Zhang Y., Liaw P.K. (2023). A review on the dynamic-mechanical behaviors of high-entropy alloys. Prog. Mater. Sci..

[9] Alvi S., Akhtar F. (2019). High temperature tribology of CuMoTaWV high entropy alloy. Wear.

[10] Günen A. (2021). Tribocorrosion behavior of boronized Co_1.19_Cr_1.86_Fe_1.30_Mn_1.39_Ni_1.05_Al_0.17_B_0.04_ high entropy alloy. Surf. Coat. Technol..

[11] Wolff-Goodrich S., Haas S., Glatzel U., Liebscher C.H. (2021). Towards superior high temperature properties in low density ferritic AlCrFeNiTi compositionally complex alloys. Acta Mater..

[12] Löbel M., Lindner T., Lampke T. (2020). High-temperature wear behaviour of AlCoCrFeNiTi_0.5_ coatings produced by HVOF. Surf. Coat. Technol..

[13] Kumar D. (2023). Recent advances in tribology of high entropy alloys: A critical review. Prog. Mater. Sci..

[14] Nagarjuna C., Dewangan S.K., Lee H., Lee K., Ahn B. (2023). Exploring the mechanical and tribological properties of AlCrFeNiTi high-entropy alloy fabricated by mechanical alloying and spark plasma sintering. Vacuum.

[15] Samoilova O., Shaburova N., Ostovari Moghaddam A., Trofimov E. (2022). Al_0.25_CoCrFeNiSi_0.6_ high entropy alloy with high hardness and improved wear resistance. Mater. Lett..

[16] Peng Z., Li B.W., Luo Z.B., Chen X.F., Tang Y., Yang G., Gong P.A. (2023). Lightweight AlCrTiV_0.5_Cu_x_ High-Entropy Alloy with Excellent Corrosion Resistance. Materials.

[17] Tikar A., Padwal S., Chen S.-H., Harimkar S.P. (2023). Analyzing the Phase Evolution, Microstructure and Wear Response of Spark Plasma Sintered Al_0.5_CoCrFeNi_2_ High Entropy Superalloy. Adv. Eng. Mater..

[18] Ragunath S., Radhika N., Aravind Krishna S., Jeyaprakash N. (2023). Enhancing microstructural, mechanical and tribological behaviour of AlSiBeTiV high entropy alloy reinforced SS410 through friction stir processing. Tribol. Int..

[19] Kasar A.K., Scalaro K., Menezes P.L. (2021). Tribological Properties of High-Entropy Alloys under Dry Conditions for a Wide Temperature Range—A Review. Materials.

[20] Menghani J., Vyas A., Patel P., Natu H., More S. (2021). Wear, erosion and corrosion behavior of laser cladded high entropy alloy coatings—A review. Mater. Today Proc..

[21] Erdoğan A., Gök M.S., Zeytin S. (2020). Analysis of the high-temperature dry sliding behavior of CoCrFeNiTi0.5Alx high-entropy alloys. Friction.

[22] Chen L.J., Bobzin K., Zhou Z., Zhao L.D., Öte M., Königstein T., Tan Z., He D.Y. (2019). Wear behavior of HVOF-sprayed Al_0.6_TiCrFeCoNi high entropy alloy coatings at different temperatures. Surf. Coat. Technol..

[23] Ersun H.B., Doleker K.M. (2022). The influence of Al addition and aluminizing process on oxidation performance of arc melted CoCrFeNi alloy. Vacuum.

[24] Joseph J., Haghdadi N., Shamlaye K., Hodgson P., Barnett M., Fabijanic D. (2018). The sliding wear behaviour of CoCrFeMnNi and AlxCoCrFeNi high entropy alloys at elevated temperatures. Wear.

[25] Yakın A., Şimşek T., Avar B., Chattopadhyay A.K., Özcan S., Şimşek T. (2022). The effect of Cr and Nb addition on the structural, morphological, and magnetic properties of the mechanically alloyed high entropy FeCoNi alloys. Appl. Phys. A.

[26] Qiu X.W., Liu C.G. (2013). Microstructure and properties of Al_2_CrFeCoCuTiNix high-entropy alloys prepared by laser cladding. J. Alloys Compd..

[27] Xiao J.K., Wu Y.Q., Chen J., Zhang C. (2020). Microstructure and tribological properties of plasma sprayed FeCoNiCrSiAlx high entropy alloy coatings. Wear.

[28] He B., Zhang N.N., Lin D.Y., Zhang Y., Dong F.Y., Li D.Y. (2017). The Phase Evolution and Property of FeCoCrNiAlTix High-Entropy Alloying Coatings on Q253 via Laser Cladding. Coatings.

[29] Daskalopoulos I., Chaskis S., Bouzouni M., Stavroulakis P., Goodall R., Papaefthymiou S. (2021). Microstructural Characterization of AlCrTiV—Si High Entropy Alloy for advanced applications. MATEC Web Conf..

[30] Qiu Y., Thomas S., Gibson M.A., Fraser H.L., Pohl K., Birbilis N. (2018). Microstructure and corrosion properties of the low-density single-phase compositionally complex alloy AlTiVCr. Corros. Sci..

[31] Feng C., Wang X.L., Yang L., Guo Y.L., Wang Y.P. (2022). High Hardness and Wear Resistance in AlCrFeNiV High-Entropy Alloy Induced by Dual-Phase Body-Centered Cubic Coupling Effects. Materials.

[32] Chauhan P., Yebaji S., Nadakuduru V.N., Shanmugasundaram T. (2020). Development of a novel light weight Al_35_Cr_14_Mg_6_Ti_35_V_10_ high entropy alloy using mechanical alloying and spark plasma sintering. J. Alloys Compd..

[33] Peng Z., Luo Z.B., Li B.W., Li J.F., Luan H.W., Gu J.L., Wu Y., Yao K.F. (2022). Microstructure and mechanical properties of lightweight AlCrTiV_0.5_Cu_x_ high-entropy alloys. Rare Met..

[34] Hao X.H., Liu H.X., Zhang X.W., Chen L., Wang Y.Y., Yang C., Liu Y. (2024). Friction–wear behaviors and microstructure of AlTiVCrNb lightweight refractory high-entropy alloy coating prepared by laser cladding on Ti–6Al–4V substrate. J. Mater. Res. Technol..

[35] Nussbaum M., Arab Pour Yazdi M., Michau A., Monsifrot E., Schuster F., Maskrot H., Billard A. (2022). Mechanical properties and high temperature oxidation resistance of (AlCrTiV)N coatings synthesized by cathodic arc deposition. Surf. Coat. Technol..

[36] Poulia A., Georgatis E., Karantzalis A. (2019). Evaluation of the Microstructural Aspects, Mechanical Properties and Dry Sliding Wear Response of MoTaNbVTi Refractory High Entropy Alloy. Met. Mater. Int..

[37] Jones M.R., Nation B.L., Wellington-Johnson J.A., Curry J.F., Kustas A.B., Lu P., Chandross M., Argibay N. (2020). Evidence of Inverse Hall-Petch Behavior and Low Friction and Wear in High Entropy Alloys. Sci. Rep..

[38] Mathiou C., Poulia A., Georgatis E., Karantzalis A.E. (2018). Microstructural features and dry—Sliding wear response of MoTaNbZrTi high entropy alloy. Mater. Chem. Phys..

[39] Qiu Y., Hu Y.J., Taylor A., Styles M.J., Marceau R.K.W., Ceguerra A.V., Gibson M.A., Liu Z.K., Fraser H.L., Birbilis N.A. (2017). lightweight single-phase AlTiVCr compositionally complex alloy. Acta Mater..

[40] Wu M.Y., Setiawan R.C., Li D.Y. (2022). Benefits of passive element Ti to the resistance of AlCrFeCoNi high-entropy alloy to corrosion and corrosive wear. Wear.

[41] Fan R., Wang L.P., Zhao L.L., Wang L., Zhao S.C., Zhang Y.J., Cui B. (2022). Synergistic effect of Nb and Mo alloying on the microstructure and mechanical properties of CoCrFeNi high entropy alloy. Mater. Sci. Eng. A.

[42] Wang W.R., Qi W., Zhang X.L., Yang X., Xie L., Li D.Y., Xiang Y.H. (2021). Superior corrosion resistance-dependent laser energy density in (CoCrFeNi)_95_Nb_5_ high entropy alloy coating fabricated by laser cladding. Int. J. Miner. Metall. Mater..

[43] Faraji A., Farvizi M., Ebadzadeh T., Kim H.S. (2022). Microstructure, wear performance, and mechanical properties of spark plasma-sintered AlCoCrFeNi high-entropy alloy after heat treatment. Intermetallics.

[44] Kong D., Guo J., Liu R.W., Zhang X.H., Song Y.P., Li Z.X., Guo F.J., Xing X.F., Xu Y., Wang W. (2019). Effect of remelting and annealing on the wear resistance of AlCoCrFeNiTi_0.5_ high entropy alloys. Intermetallics.

[45] Kadhim D.F., Koricherla M.V., Scharf T.W. (2023). Room and Elevated Temperature Sliding Friction and Wear Behavior of Al_0.3_CoFeCrNi and Al_0.3_CuFeCrNi_2_ High Entropy Alloys. Crystals.

[46] Lan L.W., Yang H.J., Guo R.P., Wang X.J., Zhang M., Liaw P.K., Qiao J.W. (2021). High-temperature sliding wear behavior of nitrided Ni_45_(CoCrFe)_40_(AlTi)_15_high-entropy alloys. Mater. Chem. Phys..

[47] Liang N.N., Wang X., Cao Y., Li Y.S., Zhu Y.T., Zhao Y.H. (2020). Effective Surface Nano-Crystallization of Ni_2_FeCoMo_0.5_V_0.2_ Medium Entropy Alloy by Rotationally Accelerated Shot Peening (RASP). Entropy.

[48] Dong T.S., Lu P.W., Ma Q.L., Li G.L., Liu Q., Fu B.G., Li J.K. (2023). Effect of laser remelting on high-temperature oxidation resistance of AlCoCrFeNi high-entropy alloy coating. Surf. Coat. Technol..

[49] Ikeuchi D., King D.J.M., Laws K.J., Knowles A.J., Aughterson R.D., Lumpkin G.R., Obbard E.G. (2019). Cr-Mo-V-W: A new refractory and transition metal high-entropy alloy system. Scr. Mater..

[50] Esmaily M., Qiu Y., Bigdeli S., Venkataraman M.B., Allanore A., Birbilis N. (2020). High-temperature oxidation behavior of AlxFeCrCoNi and AlTiVCr compositionally complex alloys, *NPJ Mat*. Degrad..

[51] Stepanov N.D., Shaysultanov D.G., Salishchev G.A., Tikhonovsky M.A. (2015). Structure and mechanical properties of a light-weight AlNbTiV high entropy alloy. Mater. Lett..

[52] Zheng Z.D., Chen Q.J., Peng X.Y., Wang H., Qu S.J., Feng A.H., Xu T., Wang K. (2023). Prediction of mechanical properties of AlTiCrVNb high entropy alloys with B2 ordered structure. J. Mater. Res. Technol..

